# Malignant Melanoma of the Gastrointestinal Tract: Symptoms, Diagnosis, and Current Treatment Options

**DOI:** 10.3390/cells10020327

**Published:** 2021-02-05

**Authors:** Darina Kohoutova, Dominic Worku, Hala Aziz, Julian Teare, Justin Weir, James Larkin

**Affiliations:** 1 The Royal Marsden Hospital NHS Foundation Trust, Fulham Road, Chelsea, London SW3 6JJ, UK; dominicworku@hotmail.co.uk (D.W.); hala.aziz@rmh.nhs.uk (H.A.); julian.teare@rmh.nhs.uk (J.T.); james.larkin@rmh.nhs.uk (J.L.); 2 Imperial College London, Hammersmith Hospital, Du Cane Road, London W12 0HS, UK; justin.weir1@nhs.net

**Keywords:** malignant melanoma, gastrointestinal tract, immunotherapy, BRAF/MEK inhibitors

## Abstract

Malignant melanoma (MM) has become the fifth most frequent cancer in the UK. It is the most common carcinoma to metastasize to the gastrointestinal (GI) tract. MM particularly has an affinity to spread to the small bowel, which is followed by the involvement of the stomach and large intestine. Excellent endoscopic options including video capsule endoscopy and enteroscopy are available for a precise diagnosis of GI involvement by a metastatic MM. The complete surgical resection of GI metastatic MM in carefully selected patients not only provides symptom control, but has also been associated with an increase in overall survival. The approval of BRAF-targeted therapies and immune checkpoint inhibitors has transformed therapeutic approaches for patients with metastatic MM over the past decade. Currently, the overall survival of patients with advanced metastatic MM who have been treated with a combination of immunotherapeutic agents reaches 52% at five years. The role of surgery for patients with the metastatic involvement of the GI tract with MM is evolving in the era of effective systemic treatments.

## 1. Introduction

Malignant melanoma (MM) is an epithelial cancer arising from melanocytes, which can be found in a range of tissue types including the eye, oral cavity, nasopharynx, anus, urinary tract, and vagina but that are overwhelmingly found in the skin [1]. While being the rarest of the skin cancers, it remains the deadliest and most likely to spread. It has now become the fifth most common cancer in the UK, with an estimated 287,700 new cases worldwide [2,3]. Our paper focuses on metastatic melanoma involving the gastrointestinal (GI) tract.

### 1.1. Secondary Malignant Melanoma of the GI Tract

Malignant melanoma is the most common carcinoma to metastasize to the gastrointestinal (GI) tract, followed by breast and lung cancer [4]. While the incidence of symptomatic GI involvement is 1–5% of MM patients, it has been demonstrated in up to 60% at post-mortem and can ultimately involve any part of the gastrointestinal tract [5]. MM particularly has an affinity to spread to the small bowel (in 51–71%), especially to the jejunum and ileum [6,7]. This might be explained by the strong melanoma cell surface expression of CCR9, a chemokine receptor, for which its CCR9 ligand, CCL25, is highly expressed in the small intestine [8]. The involvement of the small bowel is followed by metastatic spread to the stomach (27%), large intestine (22%), and the oesophagus (5%) [6]. Evidence suggests that melanoma of the extremities (15–57%) followed by the trunk (13–54%) and head and neck (5–33%) are likely to spread to the GI tract [5]. The time frame period between the diagnosis of primary MM and the identification of a gastrointestinal metastasis ranges between 2 and 180 months [9].

Patients with MM are classified by TNM classification into those with local disease (stage I–II), those with node-positive disease (stage III), and those suffering from advanced/metastatic disease (stage IV). Patients with the metastatic involvement of the GI tract have therefore stage IV disease. Current TNM staging utilizes the AJCC 8th Edition [10].

### 1.2. Primary Malignant Melanoma of the GI Tract

Interestingly, there is growing evidence of a subset of patients whereby primary melanoma of the GI tract is a possibility. It is thought that primary intestinal melanomas derive from melanoblastic cells of the neural crest which migrate via the ophalomesenteric canal to the distal ileum. Other experts believe that primary melanomas may also originate from the enteric neuroendocrine tissue of APUD cells which undergo neoplastic transformation or from neuroblastic Schwann cells of the intestinal autonomous nervous system [8,11,12,13]. Clinically, primary melanomas are diagnosed late, tend to be more aggressive, and are associated with a worse prognosis. It can be challenging to distinguish between primary melanoma and metastatic MM from unknown or regressed cutaneous origin [14].

### 1.3. Symptoms

Melanoma lesions of the GI tract are often asymptomatic but can result in a spectrum of symptoms including abdominal pain, dyspepsia, weight loss, nausea, vomiting, obstruction, perforation, acute gastrointestinal bleeding, and chronic iron-deficiency anaemia [15]. Intussusception is very rare in the adult population, representing about 5% of all cases of intussusception. The leading cause of colonic intussusception is usually a malignant lesion, yet malignant tumours are detected in only 20% of enteric intussusception cases, of which 50% are primary and 50% are metastatic [16]. Malignant melanoma has been reported as a cause of small intestinal intussusception [9,16] and also as an explanation for protein-losing enteropathy [17].

### 1.4. Diagnosis

Correct diagnosis can be made using several imaging techniques and endoscopy methods. Abdominal ultrasound, barium examinations, CT, and PET CT belong to the most commonly used imaging techniques. Abdominal ultrasound (US) is typically the first diagnostic procedure for individuals with non-specific abdominal symptoms, yet is usually not sufficient to establish a diagnosis of GI malignant melanomas. Still, the diagnosis of intestinal intussusception or extraintestinal spread including the involvement of lymph nodes can be appreciated on US [18,19]. Barium examinations (including small-bowel follow-through and conventional enteroclysis) have been standard methods for the diagnosis of intestinal MM, but they do not appreciate extraintestinal findings [18]. Cross-sectional imaging based on CT has a sensitivity of 60–70% for the detection of intestinal MM metastases [20], and an improvement in the detection rate of intestinal MM has been achieved by CT enteroclysis [18]. Whole-body PET CT has a higher sensitivity and specificity than CT scanning for all gastrointestinal metastatic MM, as demonstrated by different groups [21,22].

**Figure 1:**
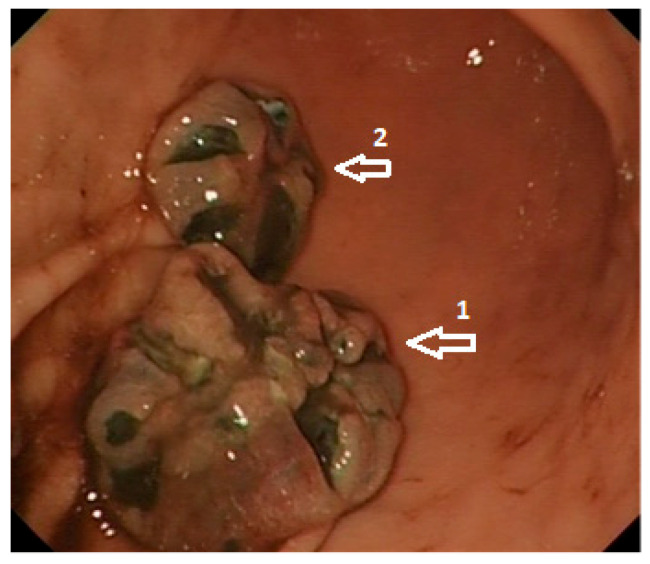
High-definition white light endoscopy: Metastatic malignant melanoma involving the distal body (size: 20 × 15 mm; 1) and proximal antrum (size: 15 × 10 mm; 2) of the stomach.

**Figure 2:**
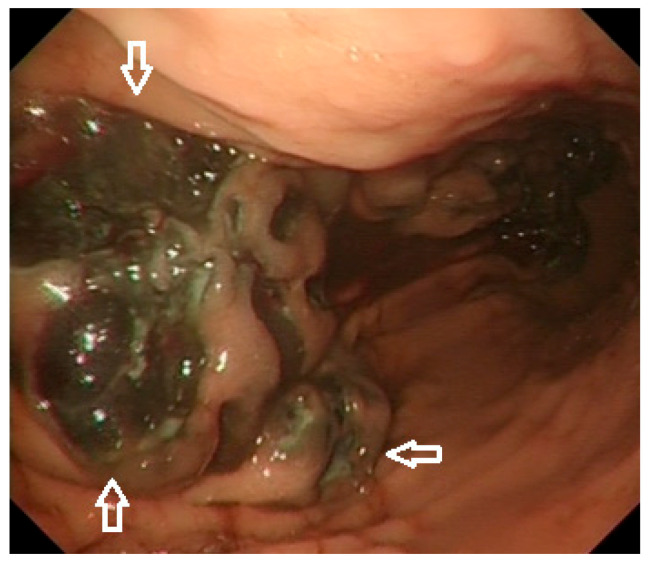
High-definition white light endoscopy: Metastatic malignant melanoma with features of recent bleeding involving the greater curvature of the gastric body (size: 30 × 20 mm; arrows).

**Figure 3:**
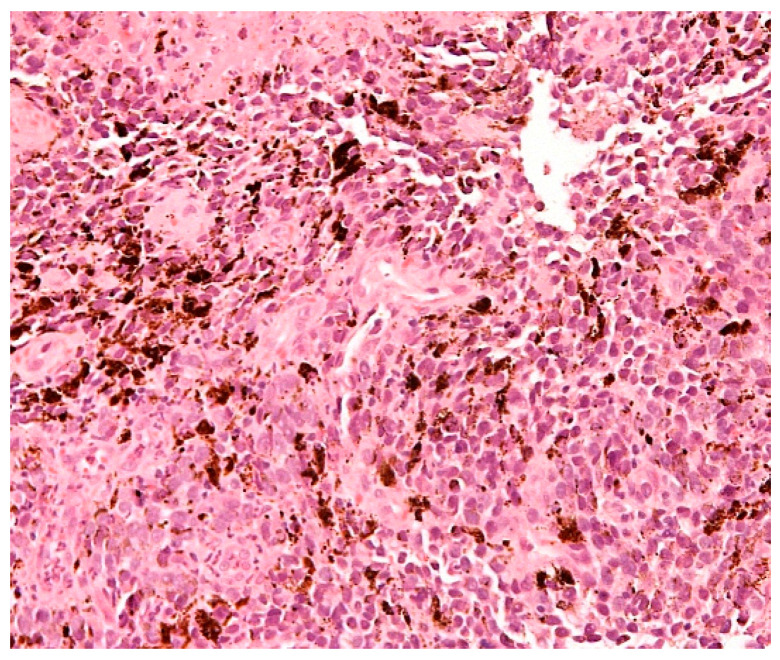
Biopsy specimen taken from the melanoma metastasis in the stomach presented in Figure 2. Haematoxyline-eosine staining (melanoma pigment—brown colour). Original magnification ×200.

Endoscopic investigations include upper and lower GI endoscopy, video capsule endoscopy (VCE), and enteroscopy. Unifocal metastatic GI lesions are more common in general; nevertheless, secondary tumours due to malignant melanoma (and also breast cancer) are typically multifocal due to their haematogenous spread [15]. Gastrointestinal MM are classified as submucosa-like or primary carcinoma-like tumours [23,24]. Lesions can be exulcerated and may present with gastrointestinal bleeding [25], as documented on our images (Figure 1 and Figure 2). Endoscopic appearances can be misleading though, as amelanotic gastric metastases have been documented [26] even in those who have had a melanotic primary [15]. Histological diagnosis is therefore required for any suspicious lesion (Figure 3).

Recently, an algorithm for the detection of small bowel melanoma metastasis was designed based on a multicentre prospective study. The study involved 390 melanoma patients of stage I–IV who were screened for signs of intestinal blood loss (either positive faecal occult blood test or overt bleeding). Independently of the signs of intestinal blood loss, all patients with stage IV disease underwent pan-intestinal endoscopy, including gastroscopy, ileo-colonoscopy, and VCE. Small bowel metastases were identified in 29% of stage IV patients, 2% of stage III individuals, and 0% of stage I/II patients. A positive faecal occult blood test was proven to be an independent negative prognostic factor for total survival in stage III and IV. The authors suggested that the VCE might be used as the first endoscopic modality, as gastroscopy and colonoscopy did not detect any additional melanoma metastasis in patients for whom VCE has not revealed any small bowel metastasis [27]. Based on the study above [27] and taking into account current clinical practice [28], we believe that the role of VCE is underestimated and this non-invasive modality should be used especially in those with stage III and IV. However, gastroscopy and ileo-colonoscopy should still be the first modalities, as gastric MM metastases are well known and patients can also suffer from anaemia and/or gastrointestinal bleeding from other sources than metastatic MM.

If a histological diagnosis is required when intestinal MM involvement cannot be confirmed based on the non-invasive VCE approach, different types of enteroscopy can be used. Push enteroscopy has been largely replaced by device-assisted enteroscopies -single- and double balloon-enteroscopies (SBE, DBE) and spiral enteroscopy. DBE was invented by Professor Yamamoto in 2001 [29] and spiral enteroscopy was introduced much later by Professor Neuhaus in 2016 [30]. Approaches have been compared recently: double-balloon enteroscopy seems to offer the deepest insertion depth to the small bowel, which is associated with the disadvantage of a longer procedural duration. Manual spiral enteroscopy seems to be a faster procedure but does not reach the depth of the DBE according to the data which are available at present [31]. Previous meta-analysis confirmed that the performance of SBE and DBE appears to be similar regarding the diagnostic and therapeutic yields, insertion depths, procedure times, complete enteroscopy, failure rates, and adverse events [32].

### 1.5. Molecular Characteristics

The main oncogenic driver mutations in cutaneous MM are the BRAF (serin/threonine protein kinase) and NRAS (neuroblastoma RAS viral oncogene) mutations, whereas KIT (tyrosine-protein kinase) mutations are predominantly observed in mucosal and acral melanomas. Around half of the patients with MM harbour BRAF mutations, of which 90% are BRAF^V600^ mutations that can be targeted with a combination of BRAF inhibitors/MEK inhibitors (MEK: mitogen-activated protein kinase inhibiting cell proliferation and inducing apoptosis) [33].

### 1.6. Treatment

#### 1.6.1. Endoscopy

Endoscopy is reserved for bleeding MM metastases. The effect can be limited; still, the following treatment options can be used and are available: adrenaline injections, haemoclips, argon plasma coagulation, and haemospray [34,35,36].

#### 1.6.2. Surgery

Multiple studies have documented that the complete surgical resection of GI metastatic MM not only provides symptom control but is also associated with a more favourable prognosis, leading to an increase in overall survival [37,38,39,40,41,42]. Ollila et al. organised a study on 124 potential surgical candidates who have been diagnosed with metastatic melanoma involving any part of the GI tract, with the exception of the oesophagus, and published the results in 1996. The study documented that 97% of the patients (67/69 operated on) experienced symptomatic relief in the postoperative period. Further, the median survival of those who underwent curative resection was 48.9 months compared to 5.4 and 5.7 months in those undergoing palliative or nonsurgical interventions [37]. A very similar survival of 47.5 months was documented for patients whose gastrointestinal metastasis was resected with curative intention in another study performed by Panagiotou at al. [39]. Berger et al. reported a shorter survival (23.5 months) for those who underwent complete resection of a GI metastasis, but this was still significantly longer compared to the survival of those with a partial resection (8.9 months); *p* < 0.0001 [40]. Curative surgery has therefore remained until now a successful intervention in very carefully selected patients with GI metastatic MM [41,43]. Most of these patients recover from the surgical procedure quickly, within six weeks [41].

#### 1.6.3. Chemotherapy

Chemotherapy is largely of historical interest, given the development of effective systemic therapy. Dacarbazine used to be the standard treatment, yet the response rate was very low, at 5–28% (average 15%) [44].

#### 1.6.4. Systemic Treatment in Advance Disease

In 2008, before the era of modern targeted therapy and immunotherapy, Korn et al. published a meta-analysis which included 2100 patients with metastatic stage IV melanoma; the median survival time was 6.2 months, with 25.5% alive at one year [45]. Over the past decade, the therapeutic landscape of MM has been revolutionised by the invention of immune check point inhibitors (ICPIs) and targeted BRAF/MEK inhibitors (BRAFi/MEKi) [46,47,48].

The discovery of ICPIs belongs to Allison and Honjo, who subsequently received the Nobel Prize in Physiology and Medicine in 2018 [49]. There are seven ICPIs known at present: (1) those targeting cytotoxic T-lymphocyte associated protein 4 (CTLA-4; ipilimumab and tramelimumab), (2) those aiming at programmed cell death receptor 1 (PD-1; nivolumab and pembrolizumab), and (3) those targeting programmed death ligand 1 (PD-L1; atezolizumab, avelumab, and durvalumab) [50].

Ipilimumab was the first treatment that showed a meaningful improvement in the overall survival of patients with metastatic melanoma. Ipilimumab was approved by the U.S. Food and Drug Administration in 2011 [51]. This was followed by the approval of vemurafenib, a BRAF^V600^ inhibitor, which lead to an improved overall survival in untreated BRAF mutant melanoma [48]. For unresectable stage III/IV, ICPIs are the current first-line standard of care treatment, regardless of BRAF status, with the use of a combination of nivolumab and ipilimumab or anti-PD1 monotherapy (nivolumab or pembrolizumab) [47,48].

Pembrolizumab was shown to be superior to ipilimumab in a randomized, controlled, phase 3 study; the 12-month survival rates were 74% (two weekly pembrolizumab), 68% (three weekly pembrolizumab), and 58% (three weekly ipilimumab) with fewer treatment-related adverse events of grade 3–5 (grade III: severe-non life threatening; grade IV: life threatening adverse event) in the pembrolizumab groups (13% and 10%) compared to (20%) the ipilimumab group [52]. Nivolumab confirmed its significant superiority to dacarbazine in previously untreated metastatic MM patients without BRAF mutation: at 12 months, the overall survival was 73% in the nivolumab arm vs. 42% in the dacarbazine arm (*p* < 0.001) [53].

Immunotherapy can be used as a single agent or in combination. Hamid et al. focused on the efficacy of pembrolizumab as a monotherapy in patients with advanced mucosal melanoma. The median overall survival was 11 months and the objective response rate was 22% and 15% in ipilimumab-naive and in ipilimumab-treated patients, respectively [54].

The landmark clinical trial CheckMate-067 looked at the efficacy of an ipilimumab/nivolumab (Ipi.Nivo) combination for advanced melanoma. The overall survival at 5 years was 52% in the Ipi.Nivo group, 44% in the nivolumab group, and 26% in the ipilimumab group. The median overall survival was more than 60.0 months (median not reached) in the Ipi.Nivo group compared to 37 and 20 months in the nivolumab and ipilimumab arms, respectively. The flattening of the survival curve indicates that half of the unresectable melanoma patients are likely to be cured from a disease that used to limit patients’ lives to 6 months 10 years ago [46].

A total of 40–50% of melanomas harbour BRAF mutations and can benefit from targeted therapy. BRAFi/MEKi are licensed in the first and subsequent treatment lines for BRAF-mutated MM [47]. A study published by Robert et al. has shown the superiority of dabrafenib (BRAF inhibitor) combined with trametinib (MEK inhibitor) to monotherapy with vemurafenib (BRAF inhibitor); the overall survival was significantly improved in previously untreated patients with metastatic MM with BRAF mutations who had received BRAF and MEK inhibitors. Combining a BRAF inhibitor with a MEK inhibitor addresses the limitations of monotherapy with a BRAF inhibitor and results in a significant delay in the emergence of resistance [55]. Another BRAFi/MEKi combination is vemurafenib and cobimetinib, which was approved following the coBRIM clinical trial performed for BRAF mutation-positive patients with unresectable or metastatic MM. The median overall survival was 22 months for patients treated with vemurafenib and cobimetinib and 17 months for those who received vemurafenib and placebo [56]. This was followed by the COLUMBUS open-label phase III trial that investigated the safety and efficacy of encorafenib (BRAFi) and binimetinib (MEKi) compared to vemurafenib or encorafenib in patients with a BRAF mutation and locally advanced, unresectable, or metastatic melanoma. Encorafenib in combination with binimetinib and encorafenib monotherapy showed a favourable efficacy compared to vemurafenib [57].

#### 1.6.5. Systemic Treatment in the Adjuvant Setting

With regard to adjuvant treatment, ICPIs or BRAFi/MEKi are currently the standard of care for surgically resected advanced melanoma based on many adjuvant clinical trials. The CHECKMATE-238 trial compared adjuvant therapy with nivolumab versus ipilimumab in those with resected stage III or IV melanoma and confirmed the superiority of nivolumab: the 12-month rate of recurrence-free survival was 71% in the nivolumab group and 61% in the ipilimumab group [58]. Nivolumab has also demonstrated a sustained efficacy benefit over ipilimumab after additional 6 months of follow-up [59]. In the adjuvant setting for resected stage III melanoma, the use of pembrolizumab was shown to lead to a significantly longer recurrence-free survival compared to a placebo [60]. Similarly, in completely resected stage III BRAF-mutated melanoma, an adjuvant combination of dabrafenib and trametinib resulted in a significantly lower risk of recurrence compared to the adjuvant use of a placebo [61].

#### 1.6.6. Combination of Surgery and Systemic Treatment in Stage IV Melanoma

The role of surgery in patients with metastatic malignant melanoma is evolving in the era of effective systemic treatment options. Smith et al. [62] compared groups of patients who underwent surgical resection for stage IV melanoma before (2003–2007) and after (2011–2015) effective systemic therapies (EST) were available. A significant difference was observed in those who underwent intra-abdominal metastasectomy: the greatest increase in the proportion of operations was observed in this subgroup of patients. Individuals with intra-abdominal metastasectomy in the era after EST had an apparently prolonged median survival compared to those before the era of EST (32 vs. 9.5 months; *p* = 0.348). Close collaboration between a medical oncologist and a surgeon with specialization in oncology is therefore crucial for the outcome of patients with metastatatic MM involvement of the GI tract. 

Summary of all outcomes is provided in Table S1.

#### 1.6.7. Side Effects of Systemic Therapies

Immunotherapy can cause immune-related adverse events in almost every organ system [63]. A retrospective study showed that the most common adverse events were colitis, hepatitis, adrenocorticotropic hormone insufficiency, and hypothyroidism, followed by type 1 diabetes, acute kidney injury, and myocarditis [64]. Skin, rheumatic toxicity and pneumonitis have also been reported [65].

The most common adverse events associated with BRAF inhibitors are cutaneous (including cutaneous squamous cell carcinoma), arthralgia, diarrhoea, fatigue, alopaecia, nausea, headaches, and pyrexia [66,67]. The most common side effects of MEK inhibitors are cutaneous (yet secondary skin neoplasms are not associated with MEKi), diarrhoea, fatigue, peripheral oedema. Additionally, cardiac, ocular adverse events as well as interstitial lung disease or pneumonitis were observed [66].

## 2. Conclusions

Metastatic involvement of the gastrointestinal (GI) tract by the malignant melanoma needs to be thought of as the majority of patients lack GI-specific symptoms. Diagnostic endoscopy tools including video-capsule endoscopy and enteroscopy are available. Until a decade ago, survival of patients with the metastatic involvement of the GI tract with MM was dismal and surgical options played the only significant role in survival and symptom improvement. The management and prognosis of this disease have been revolutionised by the current options of diagnostic endoscopy tools and the invention of targeted systemic treatment, including immune check point inhibitors and BRAF/MEK inhibitors. The combination of surgical interventions with effective systemic therapies is beneficial for this group of patients.

## Data Availability

Not applicable.

## Supplementary Materials

The following are available online at https://www.mdpi.com/2073-4409/10/2/327/s1, Table S1: Summary of all treatment outcomes.
